# Nanocarbon Catalysts: Recent Understanding Regarding the Active Sites

**DOI:** 10.1002/advs.201902126

**Published:** 2020-01-08

**Authors:** Lu‐Hua Zhang, Yumeng Shi, Ye Wang, N. Raveendran Shiju

**Affiliations:** ^1^ International Collaborative Laboratory of 2D Materials for Optoelectronics Science and Technology of Ministry of Education Engineering Technology Research Center for 2D Material Information Function Devices and Systems of Guangdong Province Institute of Microscale Optoelectronics Shenzhen University Shenzhen 518060 China; ^2^ Van't Hoff Institute for Molecular Sciences University of Amsterdam P.O. Box 94157 Amsterdam 1090GD The Netherlands; ^3^ Key Laboratory of Material Physics of Ministry of Education School of Physics and Microelectronics Zhengzhou University Zhengzhou 450052 China

**Keywords:** edge sites and topological defects, heteroatom doping, M–N*_x_*–C, nanocarbon catalysts, surface functionalization

## Abstract

Although carbon itself acts as a catalyst in various reactions, the classical carbon materials (e.g., activated carbons, carbon aerogels, carbon black, carbon fiber, etc.) usually show low activity, stability, and oxidation resistance. With the recent availability of nanocarbon catalysts, the application of carbon materials in catalysis has gained a renewed momentum. The research is concentrated on tailoring the surface chemistry of nanocarbon materials, since the pristine carbons in general are not active for heterogeneous catalysis. Surface functionalization, doping with heteroatoms, and creating defects are the most used strategies to make efficient catalysts. However, the nature of the catalytic active sites and their role in determining the activity and selectivity is still not well understood. Herein, the types of active sites reported for several mainstream nanocarbons, including carbon nanotubes, graphene‐based materials, and 3D porous nanocarbons, are summarized. Knowledge about the active sites will be beneficial for the design and synthesis of nanocarbon catalysts with improved activity, selectivity, and stability.

## Graphical abstract

The types of catalytic active sites recently reported for several nanocarbons, including carbon nanotubes, graphene‐based materials, and 3D porous nanocarbons, are reviewed.

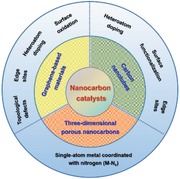

## 1 Introduction

Carbon as versatile and fascinating materials have widely been used in a great deal of technological processes, because carbon atoms can bond with each other in a variety of ways to form linear, planar, and tetrahedral bonding arrangements, thus making materials with diverse and tunable physicochemical properties.^1^ Carbon itself acts as a catalyst in various reactions, such as the aerobic oxidation of oxalic acid, the production of COCl_2_, SO_2_Cl_2_, the reductive removal of NO, and the oxidative dehydrogenation of ethylbenzene.^2^ However, the amorphous or disordered classical carbon materials (e.g., activated carbons, carbon aerogels, carbon black, carbon fiber, etc.) involved in earlier research usually showed low activity, stability, and oxidation resistance.^3^ There are three main reasons: a) traditional carbon catalysts have mainly micropores, resulting in long diffusion paths for reactant and product. Therefore, the inner active sites are utilized less. b) The random orientation and rigid crosslinking of turbostratic nanodomains by high contents of sp^3^ carbon and oxygen‐containing functionalities result in large number of different type of defective sites. As a result, it is difficult to identify the actual active sites for a special catalytic reaction. c) These carbons can also easily undergo combustion at high temperature and aerobic environment, due to the oxygen‐containing functionalities at the edges of the graphitic‐like sheet–basal planes. These factors have seriously hindered rapid development of earlier carbon catalysts.^2^

With the recent availability of nanocarbon catalysts, such as carbon nanotubes (CNTs), graphene‐based materials, and 3D porous nanocarbons, the application of carbon materials in catalysis has gained a renewed momentum. Nanocarbon materials in general have high and accessible exposed surface, which can shorten the diffusion paths, facilitating fast reaction kinetics. Therefore, the catalytic activity and selectivity of those nanocarbons are improved compared to the traditional carbons for which the reaction is limited by diffusion. The nanocarbon materials with well‐defined crystal structure are characterized by sp^2^ hybridization. Thus, these materials are more resistant to oxidation than traditional carbons when used as catalysts for high‐temperature reactions or in electrocatalysis in the presence of oxygen. However, the graphitic‐like sheet–basal planes are low free energy active sites of weak adsorption given by the stable electronic configuration of the (0001) surface orientation.^4^, ^5^ Thus, pristine carbons usually are not active for heterogeneous catalysis and surface modifications are required to make them active. Surface functionalization, doping with heteroatoms, and creating defects are the most used strategies to make efficient catalysts. However, the nature of the active sites is still not well understood. This knowledge is essential to further improve the activity, selectivity, and stability.

**Scheme 1:**
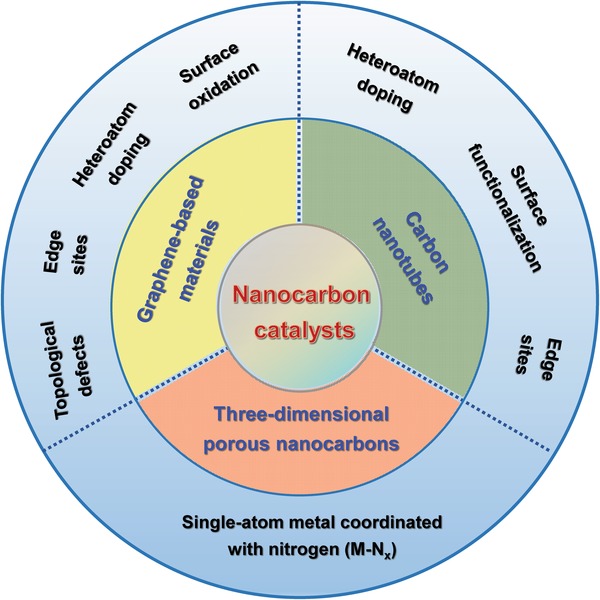
The types of catalytic active sites for three types of carbon catalysts discussed in this review.

Herein, we focus on the catalytic active sites recently reported for nanocarbon materials. Heteroatom doping is a way to incorporate various heteroatoms, such as nitrogen (N), sulfur (S), phosphorus (P), fluorine (F), and boron (B) into the carbon lattice. Because of the different electronegativities for heteroatoms and carbon atoms, the spin density and charge density of carbon atoms in the lattice are redistributed by heteroatom doping, which increases adsorption of reactants at expected sites.^6^, ^7^, ^8^ Along with the in‐depth investigation of doping effects, it is found that the appropriate doping configuration and location are more crucial than the doping content in terms of the improvement of catalytic performance. Density functional theory (DFT) simulations have revealed that the substitution of carbon atoms at graphene edge by nitrogen atoms results in the best oxygen reduction reaction (ORR) and oxygen evolution reaction (OER) activity in terms of overpotentials.^9^, ^10^ Similarly, when oxygen‐containing functionalities and vacancies are modified, the sp^2^ conjugated structure of nanocarbons will be lost. This increases active sites and thus enhances catalytic activity significantly.^11^ Besides, edge sites and topological defects are inherent and also significant in nanocarbon materials, which can tune the electronic structures of basal planes, increasing the catalytic activity. The edge sites usually include dangling groups and vacancies at the edge of the carbon. The dangling bonds located at the edge of sp^2^‐hybridized carbon basal planes usually are high‐energy sites and catalytic centers for many or redox acid‐base reactions after saturation by hydrogen or other heteroatoms. Topological defects in nanocarbon materials can occur as nonhexagonal units in the form of patterned defects or a random point mismatch.^12^, ^13^, ^14^ As described above the defects themselves catalyze the reactions, however, they are also capable of providing the unique sites for capturing metallic species. Owing to the various sizes and structures of the defects, one or more metal atoms may be trapped into the specific defect site, forming metal atom induced carbon‐defect based coordination sites, which also can catalyze the reaction. The single metal species have coordination unsaturated configurations, beneficial for highly catalytically active sites.^15^ Mostly, three mainstream nanocarbon materials including carbon nanotubes, graphene‐based materials, and 3D porous nanocarbons rich in catalytic active sites, will be discussed in detail, both theoretically and experimentally (**Scheme** 1). And a summary is finally provided together with personal perspectives on challenges and future possible trends of nanocarbon catalysts.

## 2 Carbon Nanotubes

CNTs, as representative 1D structured allotropes of nanocarbon, have attracted tremendous attention since they were firstly discovered by Iijima in 1991.^16^ CNTs possess many extraordinary mechanical, electrical, thermal, optical, and chemical properties due to its unique hollow geometry and a long‐range conjugated π electron structure, which render them ideal for wide ranges of applications.^17^, ^18^ However, pristine CNTs synthesized by the CVD method generally show a low density of functional groups with C–H terminations on the surface. Also, pristine CNTs are difficult to disperse and manipulate in many solvents. Thus, functionalization is an essential step to enhance the catalytic activity of CNTs. The functionalization improves the dispersability in aqueous solution as well as in organic solvents, leading to better catalytic activity.

The traditional methods to functionalize CNTs could be summarized to three categories: a) the covalent attachment of functional groups resulting from the reaction with π‐conjugated skeleton of CNTs; b) the noncovalent adsorption (e.g., van der Waals force, π‐stacking interactions, electrostatic force, and hydrogen bonds) or wrapping of different supermolecules; c) the endohedral filling of the inner empty cavity of CNTs. Modification of CNTs by the above approaches has been discussed in detail in some other excellent reviews.^19^, ^20^, ^21^ Herein, the latest developments in heteroatom‐doped CNTs, surface‐modified CNTs, and open‐ended CNTs will be summarized and discussed.

**Figure 1:**
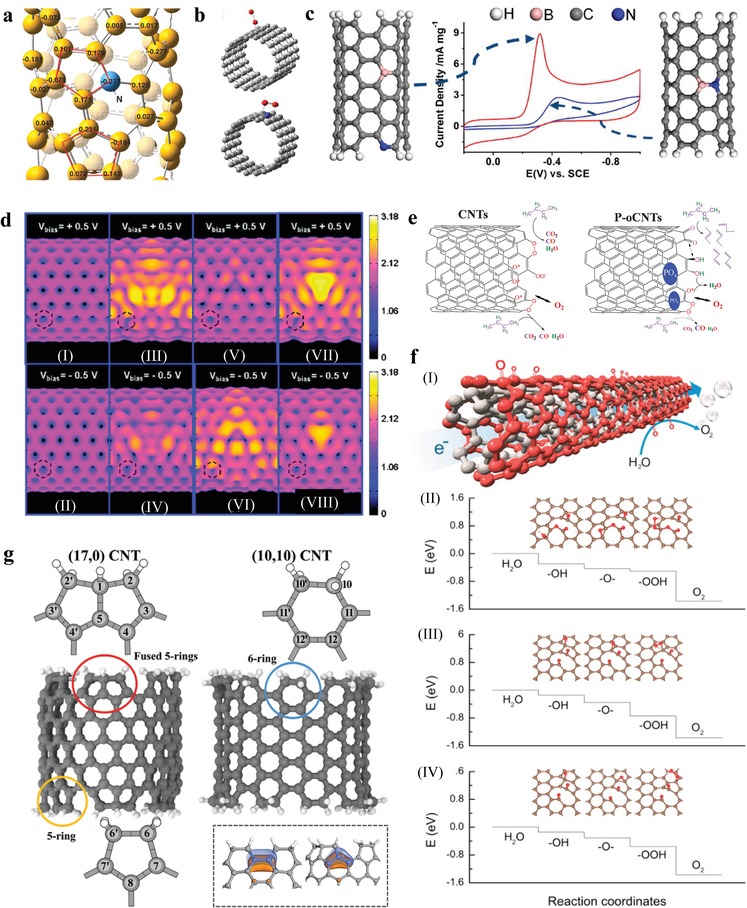
a) Calculated charge density distribution for the N‐containing CNTs. b) Schematic representing possible adsorption modes of oxygen molecules at the N‐free CNTs (top) and N‐containing CNTs (bottom). a,b) Reproduced with permission.^31^ Copyright 2009, AAAS. c) ORR performance comparison of the B and N codoped CNTs dominated by separated or bonded B and N atoms. Reproduced with permission.^37^ Copyright 2013, American Chemical Society. d) Computed STM images of pristine and doped CNTs: I,II) pristine; III,IV) pyridine; V,VI) N doping; VII,VIII) B doping. Reproduced with permission.^40^ Copyright 2010, American Chemical Society. e) Schematic reaction of butane oxidation on pristine and P‐modified CNTs. Reproduced with permission.^41^ Copyright 2008, AAAS. f‐I) A schematic illustration of OER at the surface‐oxidized MWCNTs. OER on the preoxidized graphene cluster with f‐II) both lactone and ketone groups and with f‐III,IV) ketone groups only. f) Reproduced with permission.^42^ Copyright 2015, American Chemical Society. g) Model systems of open‐ended CNTs. The (10,10) and (17,0) CNT is terminated solely by six rings, and by a mixture of carbon five rings and fused five rings, respectively. Inset: highest occupied molecular orbitals of the open‐ended (10,10) (left) and (17,0) (right) CNTs. Reproduced with permission.^45^ Copyright 2015, American Chemical Society.

Heteroatom doping has been emerged as an effective strategy in recent years to modify the surface chemistry and the electronic properties of the CNTs and thereby to promote the activity.^22^, ^23^, ^24^, ^25^, ^26^, ^27^, ^28^ N‐doped CNTs can be synthesized by CVD method using hydrocarbon as C source and ammonia or organic amine as N source.^29^, ^30^, ^31^ A good example is the enhanced catalysis by N‐doped carbon nanotube arrays in ORR shown by Dai's group. DFT simulations have revealed that the strong electronic affinity of N atoms leads to high positive charge density on the adjacent C atoms (**Figure** 1a,b), enhancing the oxygen adsorption which readily attract electrons from the anode to facilitate the ORR.^31^ Moreover, ultraviolet and X‐ray photoemission spectroscopy and spectromicroscopy as experimental results directly confirmed that N doping are effective strategy to activate the vertically aligned carbon nanotubes tips allowing the tuning of electronic properties.^32^

B atoms can also be utilized to enhance the catalytic activity of CNTs for ORR.^33^ The electron‐deficient B atoms can replace C atoms in the sp^2^ lattice. Since B has three valance electrons, they can accept electrons from C atoms, which can shift the Fermi level to the conducting band.^33^, ^34^ For the synthesis of B‐doped CNTs, organic borane is used as B source, following the same CVD process as the synthesis of N‐doped CNTs.^35^ The subsequent studies showed that codoping of CNTs leads to higher catalytic activity than monodoping.^36^, ^37^, ^38^ Notably, Hu and co‐workers revealed that not all B and N codoped CNTs show improved ORR activity (Figure 1c).^37^ Both theoretical and experimental results show that the bonded B and N cannot, while the separated B and N can activate the CNTs. The majority of the lone‐pair electrons of the N dopant are neutralized by the vacant orbital from the B dopant in case of bonded B and N. Thus, the activation of carbon *p* electrons hardly occurs owing to the lack of electrons or vacant orbitals. The separated arrangement interrupts the electroneutrality of the sp^2^ carbon to a large extent.^39^ The scanning tunneling microscopy (STM) based simulations can be used as a valuable tool to reveal the electronic properties of N and B modified graphene and CNTs. The STM images are highly relevant to the local density of states of the carbon atoms near the doping defects, providing strong theoretical assistance for future experimental research on heteroatom‐doped carbon materials (Figure 1d).^40^

Some oxygen‐containing functionalities always appear on the CNTs, because an initial oxidation process, which will produce oxygen‐containing groups and defect sites, is generally required for subsequent functionalization or doping with heteroatoms. Therefore, an understanding of the role of oxygen‐containing groups on the catalytic activity is necessary. This has also been studied recently.^41^, ^42^, ^43^ Su and co‐workers showed that CNTs with surface functional groups effectively catalyze the oxidative dehydrogenation of *n*‐butane to butenes, especially butadiene. Traces of phosphorus enhanced the selectivity significantly. The defects of bent graphitic sheets reproduce the function of oxygen heteroatoms in molecular catalysts (Figure 1e).^41^ Zhao and co‐workers also revealed that oxygen‐containing functionalities such as ketonic C=O formed on the outer wall of multiwall carbon nanotubes play crucial roles in water oxidation catalysis by changing the electronic structures of the near carbon atoms (Figure 1f).^42^ OER can only happen at the adsorption sites adjacent to the oxygen‐containing functional groups. Furthermore, Liu and co‐workers experimentally demonstrated that the carbon atoms on CNTs near the C=O functional groups are active for OER by ex situ X‐ray photoelectron spectroscopy and in situ electrochemical impedance spectroscopy.^44^

Interestingly, the recent findings of Laasonen and co‐workers suggest that pristine CNTs are not as inert as is commonly thought and that the improved catalytic activity can be obtained by the introduction of different ring structures.^45^ The catalytic sites for hydrogen evolution reaction (HER) are the surface sites introduced by the formation of different five‐ring structures at the end of the CNT, whereas the activity of traditional six‐ring sites is not greatly altered by tube termination (Figure 1g).

## 3 Graphene‐Based Materials

Graphene as a single layer of sp^2^‐hybridized carbon atoms arranged in a honeycomb lattice was discovered in 2004^46^ and has received considerable attention due to its unique physicochemical properties. The highest quality graphene is without defects in the hexagonal structure and with very low or even no oxygen content.^47^ It is a zero band gap semiconductor with the valence and conduction bands touch at the Brillouin zone corners.^48^ The zero bandgap property makes it inert for catalysis and limits its broader applications.^49^, ^50^ However, its unique structural properties make it possible to apply modification and functionalization strategies to open the bandgap, and thus broaden its application in catalysis.

Recently, Bielaswki and co‐workers used graphene oxide (GO) as a catalyst for the benzyl alcohol oxidation.^51^ This led to considerable interest in exploring GO and reduced GO's catalytic performance in more catalytic reactions, including the oxidation of sulfides and thiols,^52^, ^53^ C—H bond activation,^54^ alkylation of arenes,^55^ tandem catalysis of amines,^56^ photocatalytic hydrogen production from water,^57^, ^58^, ^59^ conversion of CO_2_ to methanol,^60^ biomass conversion,^61^ and so on.^62^, ^63^, ^64^ GO is generally obtained by exfoliation of graphite oxide synthesized by the oxidation of graphite powder with different oxidants in acidic media (Hummers method).^65^ In periphery, the GO sheets are decorated by carboxylic acid, anhydride, acetone, and phenolic groups, while the basal plane of the GO sheet is dominated by epoxy and hydroxy groups.^66^ These oxygen‐containing functionalities in graphene oxide sheets are essential for the observed catalytic activity.^67^, ^68^ When oxygen‐containing functionalities and carbon vacancies are modified, the planarity of the basal plane and pure sp^2^ configuration of GO will be lost. This increases active sites and thus enhances catalytic activity significantly.^11^ The activities of these chemically modified graphene‐based materials depend on the type and quantity of functional groups introduced. We have shown that the reaction rate and selectivity can be changed by changing the number of oxygen‐containing groups on the GO surface for the synthesis of levulinate esters from furfuryl alcohol, both are important biomass‐derived molecules.^61^

**Figure 2:**
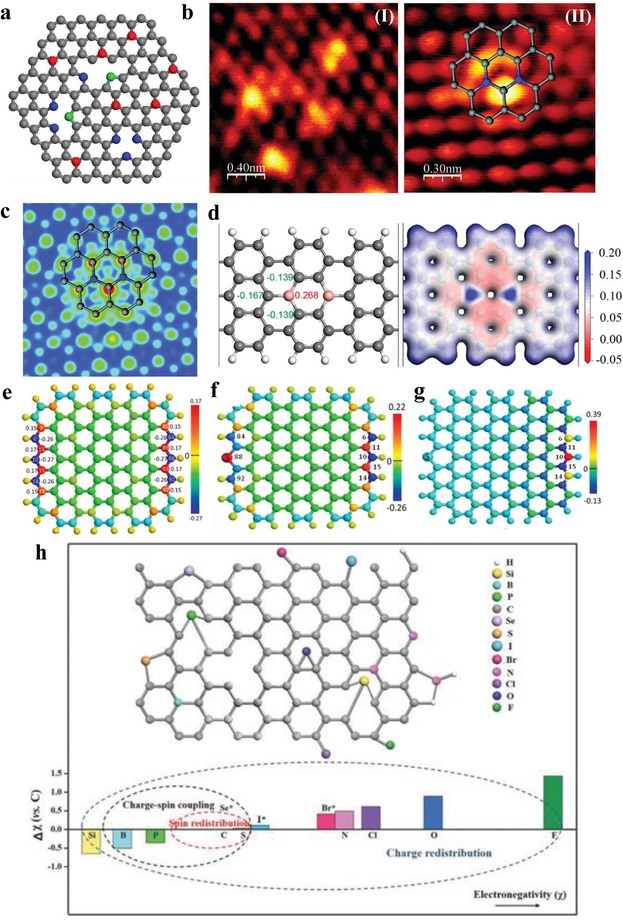
a) An atomic model for N‐doped graphene. The gray spheres represent C atoms, while the red, blue, and green ones represent graphitic, pyridinic, and pyrrolic N atoms, respectively. b) HRSTM images with defects arranged in different configurations. c) Simulated STM image for (b‐II). The inserts represent N‐doping graphene. The gray and blue spheres represent C and N atoms, respectively. a–c) Reproduced with permission.^70^ Copyright 2011, American Chemical Society. d) Top view of the B‐doping graphene cluster as well as the Mulliken charge values on B and C atoms neighbored to B atoms (left); electrostatic potential isosurface on B‐doping graphene (right). d) Reproduced with permission.^84^ Copyright 2016, American Chemical Society. e) Atomic charge density on pure graphene cluster. f) Atomic charge density and g) spin density distributions on pure graphene with S doping at the zigzag edge. The colors of sphere represent relative values of charge and spin density. The unlabeled small and large spheres represent H and C atoms, respectively. f,g) Reproduced with permission.^87^ Copyright 2014, American Chemical Society. h) Illustration of a heterodoped carbon lattice. Reproduced with permission.^8^ Copyright 2018, Wiley‐VCH.

Replacing certain carbon lattice atoms with foreign atoms (e.g., N, B, S, P, F, Cl, and Si) is also an effective method to enhance catalytic activity of graphene‐based materials. N‐doped materials are by far the most studied doped graphene materials. N can be introduced directly during the graphene growth by a CVD method in the presence of NH_3_ as the N sources^69^ or via a solvothermal process in the presence of lithium nitride and tetrachloromethane.^70^ Alternatively, N heteroatoms can also be doped through post‐treatment of graphene or GO, such as thermal annealing in ammonia,^71^, ^72^ hydrazine reduction,^73^ and the arc‐discharge method.^74^ Graphitic N, pyridinic N, and pyrrolic N with direct substitution structures are three main types of N doping.^50^ Graphitic N means that N atom replace C atom in the hexagonal rings. For pyridinic N and pyrrolic N, one and two p electrons were donated to the p system, forming sp^2^ and sp^3^ hybridized bonds (**Figure** 2a).

Based on DFT based calculations, it is well accepted that N doping can introduce asymmetry in atomic charge density and high positive spin density, modifying the electronic structures of the graphene. As research continues, numerous experimental results verify the theoretical simulations.^75^ Ferrari and co‐workers used in situ Raman measurements to monitor the dopants in an electrochemically top‐gated graphene transistor. The Raman measurements show that the G and 2D peaks show a different response to holes and electrons doping and the 2D/G height ratio changes significantly with doping, making Raman spectroscopy an ideal tool for graphene nanoelectronics.^76^ Bao and co‐workers^70^ and Ducastelle and co‐workers^77^ directly observed the distinct electronic structure perturbation induced by N doping in the graphene network via STM. As shown in Figure 2b, the bright yellow areas indicate defects, by which the graphene electronic structure was perturbed. According to the analysis, this structure perturbation likely results from the N atoms doping. The corresponding DFT simulations further supported that the neighbor C atoms show a higher brightness due to the increased electron density of states near the Fermi level introduced by N doping (Figure 2c). Schiros and co‐workers obtain a detailed picture of bond types and electronic structure in graphene doped with nitrogen at the subpercent level using photoelectron (XPS) spectroscopy, carbon and nitrogen core‐level X‐ray absorption (XAS) and emission (XES) spectroscopy in combination with DFT simulations. These findings also show that controlling over the dopant bond type is essential in advancing graphene electronics.^78^

Especially, Liu and co‐workers reported the exact function of different N species in the N monodoped carbon nanomaterials for metal‐free catalysis in different reactions for the first time.^79^, ^80^ Based on X‐ray absorption near‐edge structure (XANES) spectroscopic measurement, it was experimentally demonstrated that the electron‐donating quaternary N sites in the 3D graphene nanoribbon networks were responsible for ORR, whereas the electron‐withdrawing pyridinic N moieties in the catalyst served as active sites for OER and carbon dioxide reduction reaction.

The case of B atoms is different from that of N atoms.^81^, ^82^, ^83^ Due to the smaller electronegativity of B atoms than that of C atoms, the paired covalent electrons are slightly polarized toward C atoms, resulting local positive charge density on the B atoms (Figure 2d).^84^ Meanwhile, based on DFT calculations, a local high spin density on the basal plane could be introduced by B atom doping because of its strong electron withdrawing property, which is favorable for reactant molecules adsorption.^81^ B‐doped graphene could be obtained via thermal annealing of graphite oxide using boron oxide as the B source.^82^

Notably, the electronegativity differences between the C atoms and the foreign elements cannot be the only reason for the high catalytic activity of heteroatoms modified graphene‐based materials. For example, the electronegativities of C and S are very close (2.55 vs. 2.58), but the S‐doped graphene still exhibits excellent ORR performance.^85^, ^86^, ^87^ The improved catalytic activity in this case can be explained by spin density redistribution. DFT calculations revealed that the active sites are the carbon atoms located at the graphene edges or near the doped S and SO_2_ sites. Figure 2e,f shows that the S‐doped graphene cluster features a similar maximum charge density to that of the pure graphene (0.22 vs. 0.17), while its maximum spin density (0.39 vs. 0) was notably higher than that of the pure graphene cluster (Figure 2g). Experimentally, both benzyl disulfide^85^ and hydrogen sulfide^88^ have been chosen as S sources, leading to S‐graphene formation from GO.

Besides the modification of graphene by N, B, and S, halogenated (Cl, Br, I) graphene also shows enhanced catalytic activity.^89^ Apparently, the effects of different heteroatom doping on electronic structure of graphene can be grouped into charge‐dominated or spin‐dominated mechanisms based on electronegativity difference between foreign atoms and C atoms (Figure 2h).^8^

Since each heteroatom dopant functions via a unique mechanism, it is expected that the synergistic effect of codoping may further enhance catalytic activities of graphene‐based nanomaterials. The first example of the synergistic effect is demonstrated by B, N codoped graphene.^90^, ^91^, ^92^ Dai and co‐workers synthesized metal‐free BCN graphene simply by thermal annealing GO in the presence of boric acid and ammonia. The doping level could be adjusted, which directly influences the energy bandgap, spin density, and charge density. BCN graphene with a modest N‐ and B‐doping level (B_12_C_77_N_11_H_26_ structure) had the lowest energy gap, resulting in the best ORR electrocatalytic activity.^91^ Notably, a two‐step doping method to prepare B, N‐graphene was subsequently reported. This new method enables the incorporation of B and N atoms at desired sites on the graphene surface to induce a synergistic enhancement of the catalytic activity. DFT calculations unfold the origin of the synergistic effect: in a B–C–N configuration, the 2p orbital of the C atom is first polarized by N atom, which can then donate extra electrons to an adjacent B atom, and thus increase the electron occupancy of the 2p orbital of this “activated” B atom. As a result, a synergistic effect between B and N dopants is induced by this charge transfer process, in which electron‐withdrawing N atom indirectly activates B atom and thus make the latter an active site.^92^

In addition to B atoms, N, S codoped graphene^93^, ^94^, ^95^, ^96^, ^97^ has also been designed and synthesized. Compared to N monodoped graphene, theoretical calculations revealed that the difference of charge density around N atoms before and after S doping is similar, suggesting that the improvement of catalytic activity produced by the S doping cannot be explained by the change of electron charge density. Alternatively, difference of the spin density in codoped models is much more important than those of N monodoped ones. Therefore, the active sites could be the C atoms in the S, N codoped materials.^98^

In addition to S and B dopants, P was also doped to enhance the catalytic activity of graphene.^96^, ^99^, ^100^, ^101^ The P and N heteroatoms could affect the valence orbital energy levels of the adjacent C atom in the graphene matrix to induce a synergistically enhanced reactivity.^99^ As discussed above, codoped strategy introduced more active sites in carbon lattice, therefore incorporating more types of foreign atoms may further enhance catalytic activity of graphene‐base materials. Thus, graphene‐based materials with tridoping such as N–P–S,^102^, ^103^ N–P–F,^104^ and N–S–F,^105^ have also been synthesized and shown to improve the catalytic activity of carbon.

Edge sites and topological defects are inherent in nanocarbon materials and have attracted great attention for a long time due to their effects on electronic properties and surface reactivity.^106^ The catalytic activity of edges results from the dangling bonds that can accelerate radical chain oxidations.^59^, ^107^ Besides, edge sites can also activate covalent bonds by interacting with them.^108^ The armchair and zigzag configurations on the edges are also important. Some theoretical models reveal that the interaction of the two types of edges with small molecules like H_2_ may be different.^68^ More interestingly, carbon materials with higher edge exposure for the proper incorporation of foreign atoms, especially within the distance of armchair and zigzag configurations, are likely to be more active.^109^ Xia and co‐workers found that C atoms near the N doping on the armchair edge of graphene are the main active sites for the ORR/OER.^9^ Later works also demonstrated that O doping at defective sites and at edge sites produced the most active sites for the OER.^13^, ^14^, ^110^

**Figure 3:**
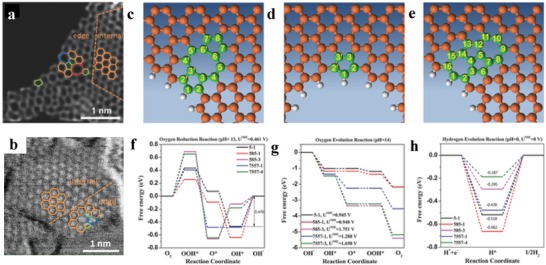
a,b) HAADF image of a 2D graphene material possessing carbon defects (DG). Hexagons, pentagons, heptagons, and octagons are marked in orange, green, blue, and red, respectively. c) Schematic structures of edge pentagon, d) 5–8–5 defects, 7–55–7 defects e) in DG. f–h) Schematic energy profiles for the ORR, OER, and HER pathway on DG. a–h) Reproduced with permission.^113^ Copyright 2016, Wiley‐VCH.

Topological defects contain intrinsic topologic vacancies and deformations at both edges and bulk domains,^111^, ^112^, ^113^, ^114^, ^115^ and are usually formed either during the synthesis process or formed naturally because of crystalline disorder. These defects in graphene materials can occur as nonhexagonal units in the form of patterned defects or a random point mismatch. Xia and co‐workers found that the charge density of C atoms near them can be significantly changed by these topological defects.^111^ The results show that the graphene clusters with line defects including pentagon–heptagon or pentagon–pentagon–octagon chains at the edges, or the point defects having pentagon rings at zigzag edge, show the catalytic activity for ORR. More recently, this prediction has been proven by experimental evidence and DFT calculations. Yao and co‐workers fabricated a dopant‐free defective graphene (DG) containing a variety of structural defects (pentagons (5), heptagons (7), and octagons (8)) with various combinations such as 585, 75585, and 5775 defect (**Figure** 3).^113^ DFT calculations suggested that the electronic structure of active C atoms was reconstructed by the edge atoms around the structural defect, and therefore their adsorption energetics were changed. C atoms adjacent to the defective edge are the active sites for oxygen related reactions. Instead, HER activity was attributed to the conjunction carbons at the defects. Wei and co‐workers also demonstrated that the N‐free graphene with adjacent pentagon and heptagon structural defects shows the lowest overpotentials for OER and ORR at the peak of volcano plots.^114^ Notably, Dai and co‐workers utilized an argon plasma etching method to produce edge/defect‐rich graphene and CNTs with improved ORR performance.^116^ These studies support the universality of the defect promoted catalysis mechanism.

## 4 3D Porous Nanocarbons

In heterogeneous catalysis, the reaction occurs on the active sites of catalyst. Therefore, the catalytic activity is expected to increase with well‐designed active sites. However, this is not sufficient to give a high apparent activity for carbon materials, because the dominant micropores of traditional carbon catalysts limit reactant and product diffusion and full accessibility to inner active sites. Engineering of 3D morphology of porous carbons with abundant accessible active sites is a promising strategy to improve the catalytic activities by offering good mass transport/diffusion paths.

Over the past ten years, there has been explosive growth in the design, synthesis, and characterization 3D porous carbon materials, such as nanocarbon sphere,^117^, ^118^, ^119^, ^120^ nanocarbon fiber,^121^, ^122^ nanocarbon sheet,^123^, ^124^, ^125^, ^126^, ^127^ and hierarchical nanoporous carbons.^128^, ^129^ Current understanding in this area allows for the synthesis of 3D porous carbons with certain micro‐/nanostructure and morphology. When used as catalysts, the 3D porous nanostructures are significantly expected to shorten the mass diffusion length, further accelerate reaction kinetics.

Most of the studies on 3D porous nanocarbon catalysts have focused on heteroatom‐doped porous nanocarbons and on single‐atom metal species coordinated defective porous nanocarbons (metal‐macrocycle complexes with different coordination environments). Currently, two strategies are usually development in the synthesis of heteroatom‐doped nanocarbons. On one hand, direct carbonization strategy is to simultaneously achieve the heteroatom doping and graphitic carbon structure formation. On the other hand, post‐treatment method is to anneal or pyrolyze the as‐prepared carbon materials with heteroatom precursors.^130^, ^131^, ^132^, ^133^, ^134^ Especially, highly N‐doped nanocarbon materials recently have drawn much attention, which provide enhanced catalytic active sites per unit mass. At present, two major strategies have been successfully developed for the synthesis of highly N‐doped nanocarbon materials. One is to develop small molecular precursors such as ionic liquids, aromatic amines, aliphatic amines, and heterocycles with high N content.^135^, ^136^ The other strategy is to construct metal–organic frameworks (MOFs) by employing small molecular precursors.^137^, ^138^ N content of nanocarbon materials prepared from MOFs precursor is as high as 29 wt%.^139^, ^140^ Besides directly catalyzing reactions, pristine and modified carbon structures can also be a good support for nanocatalysts to improve the dispersion of active metal‐related species through the enhanced affinity between carbon surface and metal precursors, and to further inhibits precursor random migration and agglomeration.^141^, ^142^

Essentially, the active sites and catalytic mechanisms of heteroatom‐doped nanocarbons and heteroatom‐doped CNTs and graphene are the same, the difference is in the morphology of materials. Therefore, we mainly review here recent progress on single‐atom metal species coordinated defective nanocarbons. Moreover, the combined experimental and theoretical studies are also discussed to bring insight into the catalytic active sites.

Such atomically distributed metal‐species‐supported catalysts have been named as single‐atom catalysts. Single‐atom catalysts contain homogenized active centers, similar to homogeneous catalysts, thus has the advantages of both homogeneous and heterogeneous catalysts. The electronic structures of single‐atom metal species can be tuned owing to diverse metal dopants and the supports, rendering them appropriate for a wide range of catalytic reactions. The electron donation from the d‐orbitals of single‐metal dopants enhance the intrinsic activity of support materials.^143^, ^144^, ^145^, ^146^, ^147^, ^148^, ^149^, ^150^, ^151^ Besides carbon‐based materials, metal oxides (sulfides),^143^, ^152^, ^153^, ^154^, ^155^, ^156^ metal nitrides,^157^, ^158^ metal surfaces,^159^ MOFs,^160^, ^161^ are also used as supports. However, carbon‐based materials, as hosts, possess the advantages such as large surface area, abundant defect sites for potential metal–support coordination.^162^ The defects including the carbon vacancy, topological defects, and N and S dopants behave as the binding sites to immobilize single‐atom metal species.

**Figure 4:**
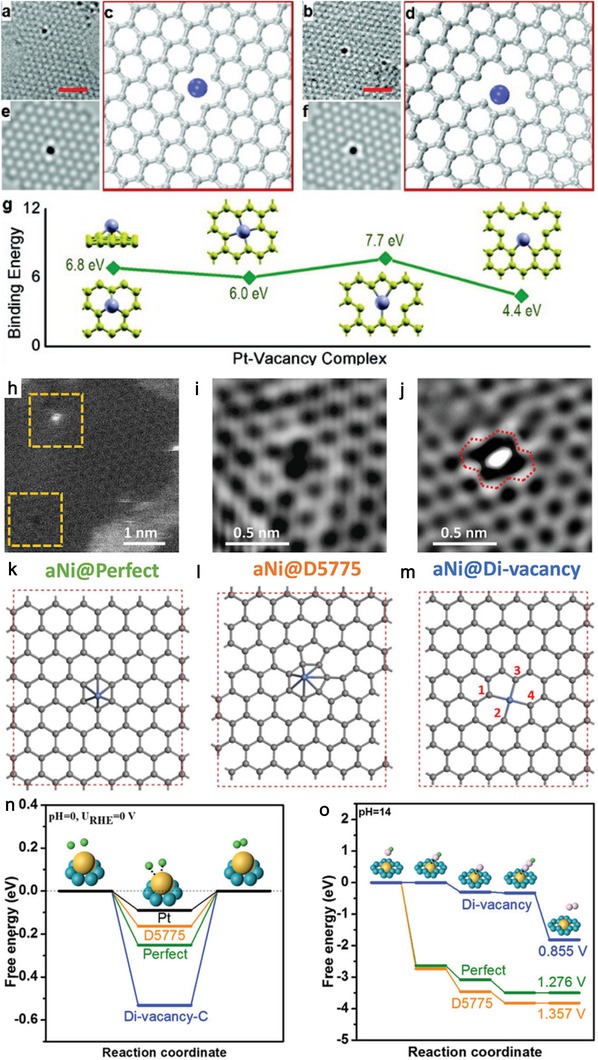
HRTEM images of a Pt atom trapped in a) a bivacancy and b) a trivacancy. c,d) Atomic models and e,f) simulated HRTEM images for the Pt–vacancy complexes in (a) and (b). g) Binding energies for different configurations. a–g) Reproduced with permission.^163^ Copyright 2012, American Chemical Society. h) HADDF‐STEM image of graphene defects with or without a Ni atom (bright white dot) in Ni@DV. i) Zoomed‐in view of bottom left box in (h). j) Zoomed‐in view of top left box in (h). Illustrations of three different types of catalytic active sites: k) aNi@defect, l) aNi@D5775, and m) aNi@divacancy. Energy profiles of the three configurations for n) HER and o) OER, respectively. h–o) Reproduced with permission.^164^ Copyright 2018, Cell Press.

Theoretically, similar to the heteroatoms, single‐atom metal species can also be regarded as foreign elements, which can modify the coordination environment of the carbon atoms. The early study on defective porous nanocarbons with coordinated single‐atom metal species was introduced by Zhang and co‐workers in 2011.^163^ They created a metal–vacancy coordination complex on the graphene by a two‐step process: first vacancies were created by high‐energy atom/ion bombardment, then these vacancies were filled with desired dopants. By this strategy, Pt, Co, and In have been successfully doped in the single‐atom form. DFT study reveals that stability of the metal–vacancy complex was ensured by its high binding energy (**Figure** 4a–g). Yao and co‐workers^164^ systematically studied the catalytic behaviors of the catalysts with defects in graphene coordinated with atomic Ni species. The catalytic active site for unique electrocatalytic reactions was assigned to a Ni atom supported on perfect hexagons (aNi@defect) revealed by DFT calculation and X‐ray absorption characterization because various local electronic densities of state of aNi (a Ni atom) were produced by the diverse defects in graphene. For example, the presence of a Ni atom supported on 5775 defect (aNi@D5775) favors the HER, while a Ni atom supported on divacancy (aNi@divacancy) is beneficial for the OER (Figure 4h–o).

Apart from providing defect sites for trapping metallic species, N, S, and O dopants in carbon materials serve as the binding sites to immobilize single‐atom transition metal species. Recently, extensive attention has been given to single‐atom metal species coordinated N‐doped nanocarbons (M–N*_x_*–C). For N‐doped nanocarbons, various N functional groups (e.g., graphitic N, pyridinic N, pyrrolic N, quaternary N, and nitrilic N) provide rich coordination sites for binding metal atoms. The binding strength between the metal and the N atoms is relatively strong because of the hybridization between the 2p‐orbitals of nitrogen and the d‐orbitals of transition metal.^151^, ^165^, ^166^, ^167^, ^168^, ^169^ Notably, the metal species themselves may not be the actual active sites and need to be coordinated with the surrounding N atoms, and the local coordination environment plays a key role for the catalysis. Moreover, the metal species are able to tune the electronic structures and configurations of the carbon, thus producing carbon catalysts with improved catalytic performance. In general, the total metal atoms incorporated into the carbon structure are relatively low, normally below 5 wt% (or 1 at%).

Initially, M–N*_x_*–C materials were synthesized by pyrolysis of carbon‐supported metal N_4_‐macrocycles precursor and used as electrocatalysts for the ORR.^170^, ^171^ During the pyrolysis process, the M–N_4_ site was partially or completely decomposed generating new functionalities and catalytic center. Recently, MOFs and ZIFs have been extensively used for the fabrication of M–N*_x_*–C catalysts.^150^, ^172^, ^173^, ^174^, ^175^, ^176^, ^177^, ^178^ In MOFs and ZIFs, metal atoms are uniformly and spatially separated by organic linkages. This unique characteristic allows metal atoms to be well isolated and captured by N atoms of carbon in the process of pyrolysis. However, it would be desirable to prepare M–N*_x_*–C catalysts from cheap precursors, such as inorganic salts, carbon, and N‐containing compounds. Along this line, different M–N*_x_*–C materials have been successfully synthesized form various inexpensive nitrogen sources such as polyaniline,^179^, ^180^ *o*‐phenylenediamine,^181^ dicyandiamide,^182^ g‐C_3_N_4_.^183^ Thus, similar to homogeneous catalyst design, the “M–N*_x_*” structures could allow the in‐depth understanding on the catalytic reaction pathways and rational design of the catalysts with tunable catalytic performance.

However, note that a variety of active sites are possible when working with M–N*_x_*–C catalysts. This raises the question about the true active site: is it single‐atom metal site, or N functionalities, or carbon defects? To answer this, carbon catalysts with exclusive metal‐defect coordination structures at atomic scales are needed. In this direction, a series of M–N_4_ coordinated carbon catalysts were prepared.^151^, ^183^, ^184^, ^185^, ^186^ Wu and co‐workers successfully made exclusive Ni–N_4_ sites on carbon for CO_2_ reduction electrocatalysis. An unprecedented high faradaic efficiency and current density for the product of CO were achieved by the Ni–N_4_ catalyst.^183^ Duan and co‐workers^185^ also prepared a series of monodispersed atomic transition metals (M = Fe, Co, Ni) trapped in a nitrogen‐doped graphene with a definitive M–N_4_–C_4_ moiety, identified by direct transmission electron microscopy imaging and systematic X‐ray absorption fine structure analyses. The catalytic activity for water oxidation of MN_4_C_4_ moieties was predicted by density functional theory and was experimentally tested electrochemically. The good catalytic activity makes M–N*_x_*–C materials a great alternative as future catalysts. Nevertheless, the stability of the M–N*_x_*–C materials is a critical issue to be resolved. Most of M–N*_x_*–C materials are stable only for a few hours, which is not enough for industrial applications. Therefore, it is critical to understand the deactivation mechanism of M–N*_x_*–C materials in order to optimize the catalyst.^187^, ^188^

Different from N species, S was usually considered as poison to metal catalysts. However, thiolated metals with the surface thiols partially removed turn out to be catalytically active, implying that the stable metal–S coordination would not completely inactivate thiolated metals.^189^, ^190^, ^191^ The subsequent research shows that the metal species was stabilized by the S‐exposing surface of nanocarbons, which can serve as coordination site.^192^, ^193^ Besides C and S atoms, O atoms on carbon supports also show strong coordination sites for metal atoms. Taking an example, Pd atoms were successfully dispersed by atomic‐layer deposition on the partially deoxygenated graphene oxide.^194^

## 5 Conclusions and Outlook

Recently, carbon catalysis has gained considerable progress because of the availability of new types of nanocarbon materials. The pristine carbons are not active for heterogeneous catalysis; thus, the research work has been concentrated on tailoring their surface chemistry. This review highlights the types of active sites that have been generally proposed as responsible for the catalytic activity of mainstream nanocarbons, including CNTs, graphene‐based materials, and 3D porous nanocarbons, in different reactions. As we have reviewed here, the active sites can be created by many methods including introduction of different ring structures as shown for CNTs, defects, heteroatom doping, immobilization of single metal atoms, or surface oxidation. The mechanisms for the enhancement of the catalytic activity also be very different. For example, for heteroatom doping, the mechanism could be either charge‐dominated or spin‐dominated. This study will be beneficial for the design and synthesis of nanocarbon catalysts with the expected performance for different catalytic reactions.

In spite of impressive progress in this field, many issues and challenges remain to be solved. First, producing a single type of active site in the carbon catalysts is still difficult. Therefore, more studies are needed to develop or improve synthesis methods to make well‐defined single‐types of active sites in various nanocarbon catalysts. Second, although the theoretical simulations are normally applied to assist the understanding of the pathways of the catalytic reactions, the in situ investigations on the catalyst surface and reaction intermediates at actual reaction conditions are still difficult. It is therefore important to develop new approaches in this direction. Third, designing and synthesizing low cost but highly active nanocarbon catalysts to speed up the practical applications is of great significance. There are indeed many efforts in this direction. As mentioned before, nitrogen‐doped carbon is possible to synthesis from cheap and abundant precursors such as melamine and glucose using simple thermal‐annealing processes. It is also possible to use edges and holes in these nanocarbons for anchoring additional catalytic sites. We showed that cooperative catalytic sites for the oxidation of ethyl lactate can be made by codoping vanadium and nitrogen on carbon.^195^ Thus, creating cooperative/synergistic effect by codoping multiple active sites can lead to highly active catalysts. We believe that considerable breakthroughs in these directions will be made in the near future so that practical catalytic applications of nanocarbons will be made possible.

Especially, carbon quantum dots, especially spherical carbon quantum dots and nanosheets like graphene quantum dots, as a new class of carbon nanomaterials have emerged recently and have garnered much interest in photocatalysis and electrocatalysis.^196^, ^197^, ^198^, ^199^ The long‐term goals in this emerging area should be to improve the synthetic production, catalytic activity, and long‐term operation stability of carbon quantum dots catalysts. Doping, surface functionalization, and carbon quantum dot‐based nanocomposites with other nanomaterials may open up new avenues for the development of carbon quantum dot‐based catalysts.

## Conflict of Interest

The authors declare no conflict of interest.
